# Mutually dependent degradation of Ama1p and Cdc20p terminates APC/C ubiquitin ligase activity at the completion of meiotic development in yeast

**DOI:** 10.1186/1747-1028-8-9

**Published:** 2013-07-01

**Authors:** Grace S Tan, Rebecca Lewandowski, Michael J Mallory, Randy Strich, Katrina F Cooper

**Affiliations:** 1 Current address: The Children Hospital of Philadelphia, Department of Pathology and Laboratory Medicine, 3501 Civic Center Boulevard, CTRB RM 4300, Philadelphia, PA, 19104, USA; 2 Department of Molecular Biology, UMDNJ-SOM, 2 Medical Center Drive,, , 08084, USA; 3 Current address: Department of Biochemistry and Biophysics, University of Pennsylvania, School of Medicine, 3700 Hamilton Walk, 19104, USA; 4 Current address: Division of Cancer Pathobiology, The Children's Hospital of Philadelphia, Colket Translational Research Building - RM 4300, 3500 Civic Center Blvd., Philadelphia, PA, 19104, USA

**Keywords:** Cdc20p, Ama1p, Anaphase Promoting Complex, Meiosis

## Abstract

**Background:**

The execution of meiotic nuclear divisions in *S. cerevisiae* is regulated by protein degradation mediated by the anaphase promoting complex/cyclosome (APC/C) ubiquitin ligase. The correct timing of APC/C activity is essential for normal chromosome segregation. During meiosis, the APC/C is activated by the association of either Cdc20p or the meiosis-specific factor Ama1p. Both Ama1p and Cdc20p are targeted for degradation as cells exit meiosis II with Cdc20p being destroyed by APC/C^Ama1^. In this study we investigated how Ama1p is down regulated at the completion of meiosis.

**Findings:**

Here we show that Ama1p is a substrate of APC/C^Cdc20^ but not APC/C^Cdh1^ in meiotic cells. Cdc20p binds Ama1p in vivo and APC/C^Cdc20^ ubiquitylates Ama1p in vitro. Ama1p ubiquitylation requires one of two degradation motifs, a D-box and a “KEN-box” like motif called GxEN. Finally, Ama1p degradation does not require its association with the APC/C via its conserved APC/C binding motifs (C-box and IR) and occurs simultaneously with APC/C^Ama1^-mediated Cdc20p degradation.

**Conclusions:**

Unlike the cyclical nature of mitotic cell division, meiosis is a linear pathway leading to the production of quiescent spores. This raises the question of how the APC/C is reset prior to spore germination. This and a previous study revealed that Cdc20p and Ama1p direct each others degradation via APC/C-dependent degradation. These findings suggest a model that the APC/C is inactivated by mutual degradation of the activators. In addition, these results support a model in which Ama1p and Cdc20p relocate to the substrate address within the APC/C cavity prior to degradation.

## Background

Meiosis is a specialized developmental program during which diploid nuclei undergo two consecutive meiotic divisions to produce haploid gametes. In the budding yeast, spore wall assembly follows the second meiotic nuclear division producing four haploid spores encased in a protective ascus [1]. Similar to differentiation programs in higher eukaryotes, meiotic progression is regulated by the transient expression of genes that are either meiosis specific or expressed during both meiotic and mitotic divisions (reviewed in [2]). In addition, progression through the meiotic divisions is also driven by the degradation of key regulatory proteins directed by the highly conserved multi-complex ubiquitin ligase called the anaphase promoting complex/cyclosome (APC/C) (reviewed in [3-6]).

During meiosis, the APC/C is sequentially activated by two of the three known Trp-Asp activator (WD40) proteins, Cdc20p (reviewed in [7,8]), and Ama1p, the latter of which is only expressed during meiosis [9,10]. The Cdc20p activated APC/C (written APC/C^Cdc20^) mediates the degradation of several key regulatory proteins including Pds1p (securin) and the S-phase cyclin Clb5 during both meiosis I (MI) and meiosis II (MII) [8,11]. Ama1p directs the ubiquitylation of the B-type cyclin Clb1p [10], Cdc20p [12] plus other unknown substrates [13] and co-ordinates exit from MII [12]. APC/C^Ama1^ also activates Smk1p, the meiotic MAP kinase required for spore wall morphogenesis [14] and is required for the early stages of spore wall assembly [11,13,15]. The third APC/C activator Cdh1p, is not required for normal meiosis [16].

It has been well documented that APC/C activator proteins recognize substrates through two conserved degrons called the “Destruction-box” (D-box, DB) and “KEN box” that bind the WD40 domain in the activator [17,18]. In addition, Doc1p (Apc10), a conserved component of the APC/C complex, also recognizes these degrons. These findings have lead to the model that substrates are recruited to the APC/C by binding to a bipartite substrate receptor composed of an activator protein and Doc1p ([19] and reviewed in [20]). During meiosis, Ama1p recognizes the D-box as well as variant of the KEN box called GxEN [10,12] whereas Cdc20p recognizes the D-box and the KEN box [21,22]. However, in *Xenopus* egg extracts the APC/C recognizes destruction motifs directly, in both a Cdc20p and Cdh1p-independent manner [23]. Similarly, much is known about how the activator proteins bind to the APC/C [5]. Structural analysis of Cdh1p has shown that a domain called the C-box interacts with Apc2p [24]. Another domain termed the IR motif promotes the association of the activator with the TPR region of several APC/C subunits (Cdc16p, Cdc23p and Cdc27p) [25-28]. Doc1p (Apc10p), a subunit of the APC/C, also associates with the TPR subunits via its IR tail [29,30]. During meiosis, both the C-box and IR domains are required for Ama1p and Cdc20p function [12]. However, mutational analysis revealed that the C-box in Ama1p is significantly more important for meiotic progression than the IR motif [12]. Similarly, during mitotic cell division, the IR box of Cdc20p is not required for function but contributes to APC/C dependent turnover [3,6].

Although much is known about how the APC/C is activated during meiotic divisions (reviewed in [8]), considerably less is known about how this ligase is inactivated as cells complete meiotic program. This is an important question as APC/C inactivation at the end of meiosis may be critical to allow the spore to reenter the mitotic cell cycle. Our previous studies have shown that both Ama1p and Cdc20p are down regulated as cells exit from meiosis II [10,12]. Furthermore, Cdc20p degradation is mediated by APC/C^Ama1^[12]. In this report, we present evidence that Ama1p down regulation occurs via ubiquitin-mediated degradation directed by APC/C^Cdc20^. Taken together, these results indicate that the cell has solved the problem of APC/C inactivation in a linear differentiation pathway by evolving a mutual degradation system for the activators.

## Results

### Cdc20p activates the APC/C to mediate Ama1p degradation

**Figure 1:**
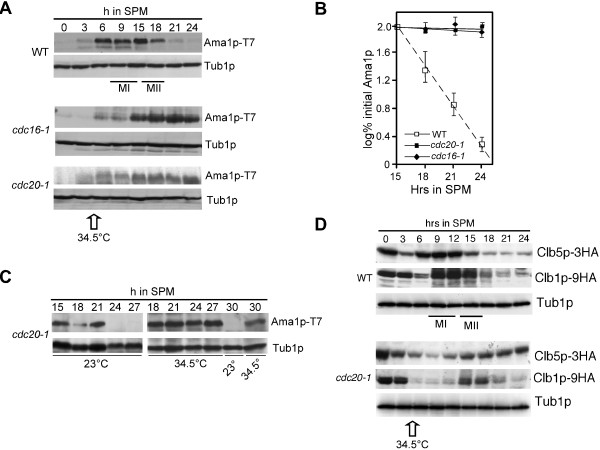
**APC/C**^**Cdc20 **^**is required for Ama1 degradation during meiosis. A**: Wild-type (RSY335), *cdc20-1* (RSY809) and c*dc16-1* strains (RSY954) harboring Ama1p-T7 (pKC3036) were induced to enter the meiosis and timepoints taken as indicated. Immunoblot analysis of immunoprecipitated protein extracts was conducted to detect Ama1p. Immunoblot analysis of Tub1p was used as a loading control. MI and MII indicate the approximate times of meiosis I (MI) and meiosis II (MII) as determined by DAPI analysis. All the strains were grown at 23°C and switched to 34.5°C (restrictive temperature for both *cdc20-1* and *cdc16-1* strains) after 4.5 h at 23°C in SPM. **B**: Quantitation of Ama1p-T7 from the experiments conducted in A. **C**: The levels of Ama1p-T7 were monitored in a *cdc20-1* strain as in Panel A except that the cells were switched to the restrictive temperature 15 h after transfer to SPM. This panel also contains analysis of Ama1p-T7 stability at both temperatures, 30 h after entering sporulation. **D**: As in Panel A except that the wild type (RSY335) and *cdc20-1* (RSY809) cultures harbored either Clb5p-3HA (pKC440) or Clb1p-9HA (pKC427) expression plasmids.

We have previously reported that Ama1p levels are reduced as cells complete the second meiotic division [10]. As APC/C activators have been reported to be down-regulated by APC/C mediated proteolysis during mitotic and meiotic cell divisions (reviewed in [7,8]), we first asked if the reduction in Ama1p levels was APC/C dependent. The meiotic levels of Ama1p-T7 [12] were monitored in a strain harboring a temperature sensitive allele of *CDC16* (*cdc16-1*), an essential component of the APC/C [31] that is required for meiosis [10]. To inactivate Cdc16-1p, the cells were switched to the restrictive temperature (34.5°C) 4.5 h after meiotic entry as previously described [8,10,32]). As a control, Ama1p degradation was also examined in identically treated wild-type cells. Immunoblot analysis revealed that Ama1p-T7 levels remained elevated in the *cdc16-1* strain compared to wild type (Figure 1A, quantitated in Figure 1B). Similar results were obtained when these experiments were repeated in a *cdc20-1* strain (Figure 1A). Furthermore, these results are consistent with those obtained when Ama1p levels were monitored in a strain where Cdc20p was inactivated during meiosis by placing it under the control of *CLB2* promoter [33]. Taken together, these results indicate that APC/C^Cdc20^ is required for the down regulation of Ama1p-T7 in meiosis.

A caveat to this interpretation is that Ama1p-T7 stabilization in the *cdc20-1* mutant is an indirect effect of the metaphase I arrest associated with this mutation [32]. To address this issue, two approaches were taken. First, we examined Ama1p stability in a *cdc20-1* mutant shifted to the restrictive temperature following meiosis II (15 h timepoint). These results show that Ama1p remains stable in the *cdc20-1* strain at restrictive temperature even following 30 h in SPM (Figure 1C). To confirm that the *cdc20-1* cells had completed the meiotic divisions by this timepoint, the transcription profiles of meiosis-specific genes were monitored using Northern blot analysis. By 15 h in SPM, maximal transcriptional accumulation of *SPS4* was observed (Additional file 1) which is an indicator that the meiotic divisions are completing [34]. Similarly, *SPS100* mRNA induction, which correlates with spore wall formation [35], occurs 18 h after meiotic entry.

For the second approach, we analyzed the meiotic degradation of Clb5p, a known substrate of APC/C^Cdc20^[11]. Clb5p-HA levels were followed by immunoblot analysis in wild type and *cdc20-1* cultures using the same temperature shift protocol as described in panel A. The results show that, compared to wild-type cells, Clb5p was stabilized following Cdc20p-1 inactivation (Figure 1D). In contrast, Clb1p, a known substrate of APC/C^Ama1^[10], is destroyed in *cdc20-1* cells using the same conditions (Figure 1D). The slower induction kinetics observed for both cyclins is due to the fact that expression of early-middle, middle gene mRNAs is significantly reduced as well as delayed in this strain background [32]. Taken together, these results support a model that APC/C^Cdc20^ mediates the degradation of Ama1p as cells complete the meiosis and begin spore morphogenesis.

### Cdh1p is not required to mediate the degradation of Ama1p during meiosis

To determine whether Cdh1p plays a role in Ama1p proteolysis during meiosis, Ama1p protein levels were monitored in *cdh1∆* cells during meiosis. The results show that *cdh1∆* cells both progress through meiosis (Additional file 2: Figure S2A, S2B and S2C) and degrade Ama1p with the same kinetics as wild type (Additional file 2: Figure S2D and see Tan et al. [12] for Northern analysis). Interestingly, dissection of the resulting *cdh1∆* tetrads revealed that, different to previously published results [16], *cdh1∆* spores exhibit a significant reduction in their ability to form colonies (Additional file 2: Figure S2E). These results indicate that Cdh1p does not control Ama1p stability but does play a role in promoting spore viability.

### Ama1p contains functional degradation signals

**Figure 2:**
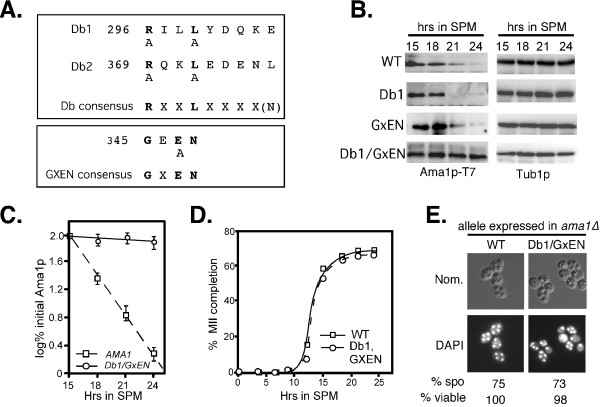
**Identification of Ama1p degrons. A**: Location of conserved APC/C degrons in Ama1p. The consensus sequences of destruction box and GXEN motifs are in bold face. The mutations described in the text are indicated below the consensus sequences. **B**: Both Db1 and GxEN degrons mediate Ama1p degradation during meiosis. Wild-type cells (RSY335) harboring plasmids expressing Ama1p-T7 or mutants as indicated were induced to enter meiosis and samples taken for immunoprecipitation and immunoblot analysis at the timepoints indicated. Tub1p levels were used as a loading control. **C**: Quantitation of the degradation kinetics of wild-type Ama1p-T7 and the Db1-GxEN double mutant obtained in Panel B. The mean ± s.e.m. is shown for each timepoint (n=3 independent experiments). **D**: The percent of tetra-nucleated cells during a meiotic timecourse in *ama1∆* cells (RSY562) expressing either wild-type Ama1p (squares) or the DB1/GxEN double mutant (circles) plasmids. **E**: Fluorescence microscopy (1000X magnification) and Nomarski optics (Nom.) of DAPI ^Db1/GxEN^ expression plasmids. The percent viability of dissected spores (n=40, WT normalized to 100%) is given below.

Ama1p contains two motifs, the destruction box (Db) and GxEN, that are recognized by APC/C^Cdc20^ (reviewed in [36]), see Figure 2A). To determine if these sequences are required for Ama1p-T7 degradation, wild-type cells expressing either Ama1p^Db1∆^-T7 or Ama1p^GxEN^-T7 mutant proteins were induced to enter meiosis and their degradation profiles monitored by immunoblot analysis. These studies revealed no difference in decay kinetics for the single mutant derivatives compared to wild type (Figure 2B) indicating that individually the Db1 or GxEN motifs are not essential for Ama1p degradation. We have recently shown that the APC/C^Ama1^ mediates Cdc20p degradation through more than one degron [12]. To determine if Cdc20p also recognizes multiple Ama1p degrons, wild-type cells expressing a double Db1 and GxEN *AMA1* derivative were examined as just described. The results (Figure 2B, quantified in Figure 2C) show that combining the GxEN and Db1 mutations protected Ama1p-T7 from degradation similar to that observed in *cdc16-1* cells (compare to Figure 1A). These results indicate that either Db1 or GxEN is sufficient to target Ama1p for degradation. No difference in the rate of meiotic progression (Figure 2D) or spore viability (Figure 2E) was noted indicating that stabilizing Ama1p did not have an adverse effect on the process.

### Ama1p is a substrate of APC/C^Cdc20^ in vitro

**Figure 3:**
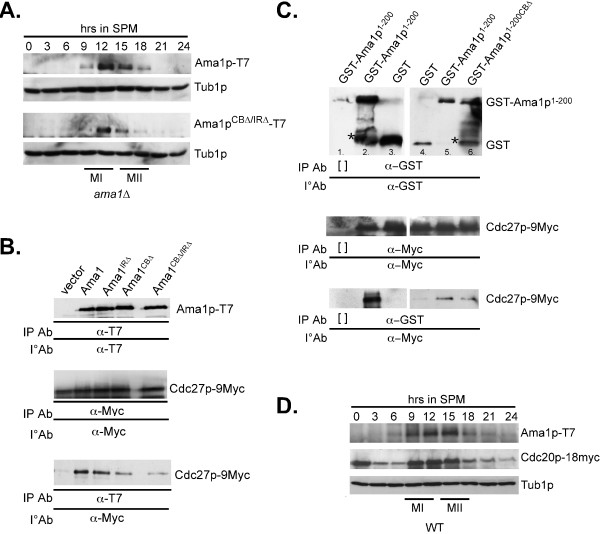
**Ama1p binding to the APC/C is not required for its degradation. A**: *ama1∆* strain (RSY562) harboring either Ama1p-T7 (pKC3036) or Ama1p^CB∆/IR∆^-T7 (pKC3048) expression plasmids were induced to enter meiosis and timepoints taken as indicated. Immunoprecipitation and immunoblot analysis of protein extracts was conducted to detect Ama1p-T7 and Ama1p^CB∆/IR∆^-T7. Immunoblot analysis of Tub1p was used as a loading control. **B**: Ama1p deleted for the CB and IR regions shows reduced binding to Cdc23p-9myc during meiosis. The Cdc27-9myc expressing strain (KCY328) harboring either the vector control, Ama1p-T7 or mutant versions of Ama1p as indicated were induced to enter meiosis and the cells harvested 12 h following transfer to SPM when both *CDC27* and *AMA1* are expressed. Immunoprecipitation and immunoblot analysis was conducted to detect the presence of both proteins. The top and middle panels control for protein expression (input). The bottom panel assays co-immunoprecipitation. **C**: The amino-terminal region (codons 1-200) of Ama1p is sufficient for APC/C association. The Cdc27-9myc expressing strain RSY1337 harboring either GST (lanes 3 and 4), GST-Ama1p^1-200^ (lanes 1, 2 and 5) or GST-Ama1p^1-200CB∆^ (lane 6) expression plasmids were grown in raffinose/galactose medium to induce the fusion genes. Immunoprecipitation and immunoblot analysis was conducted to detect the presence of both proteins. The top and middle panels control for protein expression (input). The bottom panel assays co-immunoprecipitation. [] represents the no antibody mock immunoprecipitation. The asterisk represents a background band. **D**: A wild-type strain (RSY750) harboring integrated *AMA1*-3HA and *CDC20*-18myc alleles were induced to enter meiosis and timepoints taken as indicated. Immunoblot analysis of immunoprecipitated protein extracts was conducted to detect Ama1p-3HA and Cdc20p-18myc. Immunoblot analysis of Tub1p was used as a loading control. In all experiments, the approximate times of meiosis I (MI) and meiosis II (MII) were determined by DAPI analysis.

**Figure 4:**
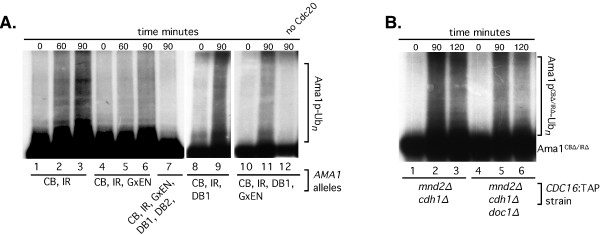
**Ama1p ubiquitylation by APC/C**^**Cdc20 **^**A: in vitro ubiquitylation of Ama1p and mutant derivatives as indicated using the APC/C prepared from *mnd2∆ cdh1∆ CDC16*::TAP strain (RSY1381, see Methods for details).** In vitro transcription coupled translation produced Cdc20p was added to all extracts except for lane 12. ^35^S labeled Ama1p harboring the following mutations:- lanes 1, 2 and 3 CB∆/IR∆, lanes 4, 5 and 6 CB∆/IR∆/GxEN, lane 7 CB∆/IR∆/GxEN/Db1/Db2, lanes 8 and 9 CB∆/IR∆/Db1 and lanes 10,11 and 12 CB∆/IR∆/GxEN/Db1 was prepared by in vitro transcription coupled translation. **B**: Doc1p is not required for APC/C^Cdc20^ mediated ubiquitylation of Ama1p. In vitro ubiquitylation assays on Ama1p^CB∆/IR∆^ using APC/C purified from *mnd2∆, cdh1∆ CDC16*::TAP (RSY1381, lanes 1, 2 and 3) or *mnd2∆ cdh1∆ doc1∆ CDC16*::TAP (RSY1748 lanes 4, 5 and 6). Time after the addition of Cdc20p to the reactions (minutes at 37°C) is given.

To further confirm that APC/C^Cdc20^ mediates the degradation of Ama1p, in vitro ubiquitylation assays were performed (see Methods for details). As Ama1p is an activator of the APC/C [10], the assays were performed with an in vitro transcription coupled translation produced 35-S labeled Ama1p derivative deleted for its two APC/C binding domains (C-box and IR motif). These motifs are required for Ama1p function [12] and their mutation reduces its association with the APC/C (see Figure 3B and C). To ensure that the added Cdc20p is the only activator in the reaction, the APC/C core complex was purified from mitotically dividing *cdh1∆* cells. Furthermore, Mnd2p (Apc15p) was not present in the extracts as it inhibits meiotic APC/C activity [33]. As predicted from the in vivo studies, Ama1p^CB∆/IR∆^ is ubiquitylated by APC/C^Cdc20^ in vitro (Figure 4A, lanes 1, 2 and 3 and see Additional file 3 for input), but also that Cdc20p is required for this event (Figure 4A – lane 12).

The in vivo stability assays just described (Figure 2) indicated that either Db1 or the GxEN motif is sufficient to induce Ama1p degradation. Consistent with this result, deletion of either of these motifs in the Ama1p^CB/IR^ mutant still allowed ubiquitylation to occur (Figure 4A, lanes 4-6 for GxEN, 8 and 9 for Db1). However, Ama1p mutated for both Db1 and GxEN was still ubiquitylated in vitro by APC/C^Cdc20^ (Figure 4A, lanes 10 and 11). This result was unexpected as this mutant is not targeted for degradation in vivo (Figure 2B). These results led us to test if the second destruction box degron (Db2) on Ama1p can mediate Cdc20p-dependent in vitro ubiquitylation. This was indeed the case as the mutation of Db2, in addition to Db1 and GxEN, rendered Ama1p resistant to APC/C^Cdc20^-dependent ubiquitylation (Figure 4A, lane7). Taken together, these results reveal that Cdc20p can recognize degrons Db1, Db2 and GxEN using in vitro assays. However, Db2 is not recognized by Cdc20p as a degron in vivo during meiosis.

The APC/C core component Doc1p forms part of the bipartite degron receptor in yeast [19,25,30]. Therefore, we addressed whether Doc1p is required for APC/C^Cd20^ mediated ubiquitylation of Ama1p. The ubiquitylation assays were repeated using Ama1p^C-Box∆/IR∆^ as the substrate and APC/C was prepared from *cdh1∆ mnd2∆ doc1∆* cells. The results show a slight qualitative reduction in Ama1p^C-Box∆/IR∆^ ubiquitylation when the APC/C was prepared from *cdh1∆ mnd2∆ doc1∆* extracts compared to those prepared from a *cdh1∆ mnd2∆* strain (Figure 4B, compare lane 3 to 6). These results suggest that Doc1p is dispensable for Ama1p ubiquitylation in vitro.

### Ama1p association with the APC/C through its C-box and IR motif is not required for its degradation

Significant structural analysis of the APC/C and its substrates has found two distinct locations within the cavity of the core APC/C complex that are occupied by the activator protein and the substrate. Our findings that Ama1p is both an activator and a substrate of the APC/C raised the question of its location within the APC/C cavity before it was destroyed. To address this question, we took advantage of the observation that the conserved APC/C binding domains of Ama1p (C-box and IR motif) are required for APC/C^Ama1^ function and normal association with the APC/C [12]. Therefore, we reasoned that if Ama1p was destroyed while in its activator binding pocket, then disruption of this interaction should protect the protein from degradation. Immunoblot blot analysis of *ama1∆* cells harboring either wild-type Ama1p or Ama1p^CB∆/IR∆^-T7 during meiosis revealed no differences in the kinetic profile of Ama1p accumulation and degradation (Figure 3A). These results indicate that Ama1p association to the APC/C via the CB and IR motifs is not a pre-requisite for its degradation. These results also suggest that the majority of Ama1p degradation is not mediated by auto-ubiquitylation as Ama1pCB∆^/IR∆^-T7 is still degraded in the absence of a functional copy of Ama1p.

To further address this question, co-immunoprecipitation performed assays were performed between Cdc27p-9myc and either Ama1p, Ama1p^CB∆^-T7, ^Ama1pIR∆^-T7, or Ama1p^CB∆/IR∆^-T7. The results showed that Ama1p^CB∆^-T7 and Ama1p^CB∆/IR∆^-T7, which complemented an *ama1∆* allele with 11 and <0.5% sporulation efficiency, respectively [12], ^exhibited^ reduced Cdc27p-9myc binding (Figure 3B). Conversely, Ama1p^IR∆^-T7, which exhibited only slight reduction in activity [12], binds Cdc27p-9myc with similar affinity as wild-type Ama1p. These results were somewhat unexpected as deleting the IR and Cbox motifs in Cdh1p eliminates its ability to bind the APC/C [37]. In addition, these results suggest the presence of additional APC/C binding motif(s) in Ama1p. Consistent with this possibility, we found that a GST-Ama1p fusion construct containing the divergent amino third of Ama1p (codons 1-200) [12], can co-immunoprecipitate with Cdc27p-9myc (Figure 3C) whereas GST alone cannot (lanes 3 and 4). Again, we only observe a slight reduction in Cdc27p-9myc association when a GST-Ama1p^1-200CB∆^ fusion construct (Figure 3C, lane 6). These results indicate that the amino-terminal region of Ama1p is sufficient for APC/C association and contains an uncharacterized APC/C binding motif(s).

### Cdc20p and Ama1p are degraded with the same kinetics during meiosis

We have previously demonstrated that APC/C^Ama1^ directs the degradation of meiotic Cdc20p [12]. Our results here indicate that in a reciprocal fashion APC/C^Cdc20^ also mediates the degradation of Ama1p as cells exit meiosis II. If Ama1p and Cdc20p are required for each other’s degradation, one prediction of this model is that their degradation kinetics should be similar. To test this hypothesis, a strain was constructed harboring integrated alleles of *CDC20-*18myc and *AMA1*-3HA under the control of their own promoters. Our previous studies found that Ama1p-3HA is both functional and has the same degradation kinetics as Ama1p-T7 [10]. A meiotic timecourse was conducted and Cdc20p-18myc and Ama1p-3HA expression profiles were determined by immunoblot blot analysis. These studies revealed that the accumulation and subsequent degradation of both proteins were remarkably similar (Figure 3D). These results are consistent with the model that Ama1p and Cdc20p simultaneously mediate each other’s degradation, thus terminating APC/C activity as the cells complete meiosis and form quiescent spores.

## Conclusions

The APC/C ubiquitin ligase is required for the meiotic nuclear divisions in yeast. Previous studies have found that the two APC/C activators in meiosis, Ama1p and Cdc20p, are down regulated as cells complete meiosis II. Cdc20p is targeted for degradation by APC/C^Ama1^[12]. In this study, we demonstrate that the reverse is true in that APC/C^Cdc20^ is required for Ama1p degradation. Using a combination of stability assays and in vitro ubiquitylation experiments, we show that Cdc20p, but not Cdh1p, targets Ama1p through either one of two degrons, Db1 and GxEN. We also provide evidence to support a model in which degradation of Ama1p does not occur by auto-ubiquitylation as the non-functional Ama1p^CB∆/IR∆^ mutant is still degraded with wild-type kinetics in *ama1∆* cells. Finally, we show that the degradation of Ama1p and Cdc20p at MII exit occurs with similar kinetics. Taken together, these results suggest a model in which the mutually dependent degradation of Ama1p and Cdc20p terminates APC/C ubiquitin ligase activity at the completion of meiotic development in yeast.

Understanding how the APC/C is regulated during both mitotic and meiotic divisions is important as unscheduled APC/C activity can lead to mis-segregated chromosomes and aneuploid gametes. Many studies have been devoted dissecting the precise mechanisms by which the APC/C is both activated and inactivated in mitotic cells (reviewed in [5]). These studies revealed that the complete inactivation of the APC/C late in G1 is driven by inhibition of Cdc20p and Cdh1p. This system not only resets the APC/C clock, which is critical for maintaining ploidy as it ensures that the pre-replication complex is assembled prior to S phase (reviewed in [36]). Cdh1p inactivation is achieved by phosphorylation (reviewed in [7]). However, Cdc20p regulation is more complex. Initially, it was shown that Cdc20p is inactivated by transcriptional oscillation and turnover by APC/C^Cdh1^ (reviewed in [4]). However, recently it was shown that APC/C^Cdh1^ only partially contributes to Cdc20p degradation during anaphase [38]. Instead, Cdc20p degradation is predominantly mediated by an auto-ubiquitylation event [6,39]. Ama1p degradation does not seem to take the same course as the non-functional CB∆/IR∆ is still degraded in *ama1∆* cells (Figure 4A).

Even less is known about how the APC/C is inactivated as cells exit meiosis II. This is an important question as APC/C inactivation is important for normal embryonic development in *Drosophila*[40]. Similarly, we find that the two APC/C activators are degraded late in meiotic development. However, we find no significant effect on meiosis II fidelity or overall spore viability when either Cdc20p or Ama1p degradation is inhibited ([12] and Figure 2). These observations suggest that either APC/C inactivation is not required for the normal execution of meiosis and spore formation or that this ubiquitin ligase is disabled by redundant systems. In support of the latter possibility, several mechanisms are known to control APC/C function including inhibitory phosphorylation [41-44], APC/C specific inhibitors [45-52], or removal of the activator from the APC/C complex [53]. The roles these mechanisms play as cells exit the meiotic program are not well understood. However, in *Xenopus* and *S. pombe*, inhibitors of meiotic Cdc20p have been identified [54,55].

### Model for substrate recognition by APC/C activators

**Figure 5:**
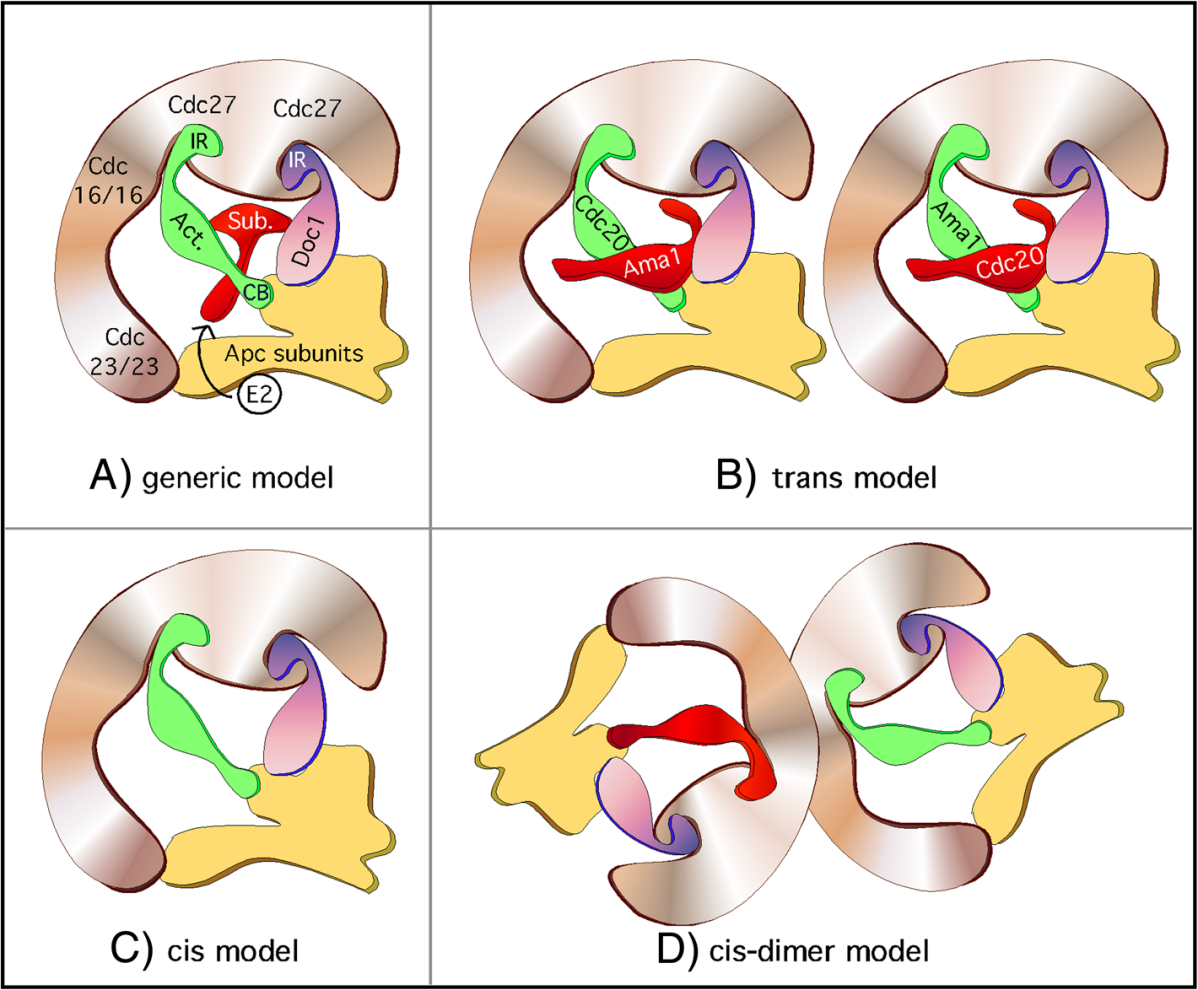
**Possible mechanisms for mutually dependent degradation of Cdc20p and Ama1p. A***:* Generic APC/C model derived from genetic, biochemical and structural information (adapted from models presented in [19,24,58,59]); both the activator (green) and the substrate (red) are located in the inner cavity of the multi-subunit complex. The substrate is represented as binding between the interface of the activator (via D-box or GxEN) and Doc1p (purple, via D-box [30]). The “platform” (Apc1p, Apc4p and Apc5p) and Apc2p are shown in blue and the “arc lamp” (Cdc16p, Cdc23p and Cdc27p) in light brown. The activator is connected to the arc lamp (via Cdc27p) and to the platform (via Apc2p) by its IR and C-box motifs respectively [67]. Doc1p is also connected to Cdc27p via its IR motif and to Apc2p (reviewed in [3,5]). E2 shuttles into the complex during the course of a polyubiquitylation reaction. **B**: Trans-model. Ama1p and Cdc20p are destroyed when they are released from the activator binding position and move into the substrate position. **C**: Cis-model**.** Ama1p remains in the activator position and is destroyed by auto-ubiquitylation. **D**: Cis-dimer model. Ama1p and Cdc20p remain in the activator position. They are destroyed when they come in contact with another APC/C subunit bearing the reciprocal activator.

Extensive studies have been devoted to understanding the molecular mechanisms of APC/C activator binding and substrate recognition (reviewed in [5]). Currently, two non-mutually exclusive models have been proposed. In the bi-partite model (outlined in model A, Figure 5), the substrate binds to both the activator and to Doc1p in the inner cavity of the APC/C. This dual association increases the affinity of the substrate enzyme complex [19,24,25,30]. However, Doc1p it is not essential for substrate binding in yeast [56] and its contribution to meiosis is not well documented. In the second model, coined the allosteric model, binding of the activators to the APC/C induces a conformational change which leads to substrate recognition [57]. Currently, the bipartite model is favored but the two models can co-exist as the bi-partite model can still accommodate activator association promoting conformational changes.

That being said, how does Ama1p fit into these models when it becomes a substrate of the APC/C? Recently, work by Foe *et al*. [6] has shed some light on this question. This group demonstrated that the majority of the late mitotic turnover of Cdc20p occurs while Cdc20p is bound as an activator and is driven by auto-ubiquitylation (see model in Figure 5C, cis-model). Consistent with this model, Cdc20p^IR∆^ mutants show increased steady state levels and reduced auto-ubiquitylation [3,6]. In contrast, we present evidence that Ama1p degradation is independent of APC/C binding via the CB and/or IR motifs (see Figures 4 and 3A). As the CB and IR motifs associate with Cdc27p/Cdc23p and Apc2p, respectively [3], our data support a model (outlined in Figure 5B, trans-model) in which Ama1p disassociates from Cdc27/23 and Apc2 before it is recognized as a substrate by APC/C^Cdc20^. Thus, the residual association that we observed between Cdc27p and Ama1p^CB∆/IR∆^ (Figure 3B and C) could be due to Ama1p associating with the APC/C in the substrate location. This suggests a model in which C-box and IR motifs anchor Ama1p in the activator position but in their absence, Ama1p switches into the substrate position binding the APC/C via as yet uncharacterized motifs. The mechanism that triggers this disassociation remains unknown but recently it has been shown that phosphorylation of Cdc20p prevents its CB-dependent activation of the APC/C in *Xenopus* egg extracts [44]. Lastly a “cis-dimer” model (Figure 5D) where Ama1p remains in the activator position and is degraded when an APC/C^Cdc20^ complex forms a dimer partner is also possible. This model is not favored as although yeast APC/C exist as dimers, recent work has shown that the monomers associate along the backbone of the “arc lamp” thus positioning the substrate binding sites in opposite directions [19,60].

Finally, the observation that Cdc20p and Ama1p both regulate each other leads to the mechanistic question of which protein is the last one to be degraded. Analysis of both proteins under the control of their own promoters in a single meiotic timecourse experiment showed that they were down regulated at the same time. These results suggest that it may not be critical as to which activated APC/C molecule is the last one. To conclude, these data presented here allow us to propose a model of how APC/C activators are recognized as substrates of the APC/C during meiosis. It remains to be seen if this model is conserved during gametogenesis in other systems.

## Methods

### Yeast strains and plasmids

The strains used in this study (Table 1) are isogenic to RSY335 [61] and are derived from an SK1 background [62]. The only exception to this is RSY1337 that is isogenic a W303a-related strain RSY10 [63]. The Cdc27-9myc::*LEU2* strains (KCY328 and RSY1337) were made by inserting *CDC27*-9myc tagged allele (P. Hieter) into RSY335 and RSY10 respectively. The *mnd2∆::*KANMX *cdh1∆*::*LEU2 CDC16*-TAP strain (KCY1381) was made as follows. First, the TAP cassette was inserted into the carboxyl terminus of *CDC16* by recombining PCR products from pFA6a-TAP-kanMX6 (D. Barford) to create KCY456. Next, the *mnd2∆::*KANMX haploid (KCY419) was created in the opposite mating type using the gene disruption [64]. These two haploids were then mated and an *mnd2∆::KAN CDC16::TAP::*KANMX haploid (RSY1248) spore clone was identified that showed 2:2 distribution of the KANMX allele following tetrad analysis. *CDH1* was deleted from RSY1248 using pWS176 (W. Seufert) to create RSY1381. Finally *DOC1* was deleted from this strain using standard gene disruption techniques [64] to create RSY1748. The temperature-sensitive *cdc20-1* strain (RSY809) has been previously described [32]*.* The temperature-sensitive *cdc16-1* strain RSY954 was made by back crossing H20c1a5 [10] into the RSY335 strain background eight times. The strain harboring integrated epitope-tagged alleles of both *AMA1* and *CDC20* (RSY750) was made by using integrating plasmids containing functional *AMA1*-3HA [10] and *CDC20*-18myc (from W. Zachariae), respectively. Tables 2 and 3 list the oligonucleotides and plasmids used in this study, respectively. Details of plasmid constructions are available on request. In brief, all the *AMA1*-T7 tagged plasmids were derived from pKC3036 [12]. The Ama1p expressing plasmids used for ubiquitylation assays were derived from pME67 (D. Morgan). The Cdc20p plasmid used for ubiquitylation assays was pME41 (D. Morgan). The *CLB5*-3HA plasmid (pKC440) was made by cloning an *Xho1*-*Cla*1 fragment containing Clb5-3HA (from C. Wittenberg) under the control of its own promotor and terminator into Ycplac222. The Clb1-9HA plasmid was made by first cloning a *Pst*1-*Pst*1 fragment from a *CLB1*/*CLB6* contig (from C. Wittenburg) into pRS315 and then inserting 9 repeats of the HA epitope just upstream of the stop codon to create pKC427. The galactose inducible GST-Ama1^1-200^ fusion construct (pKC3113) has been previously described [12]. In brief, *AMA1* was introduced into pEG[KT], which contains GST under the control of the galactose promotor (a gift from M. Solomon). Site directed mutagenesis was used to delete the C-box in this construct to make pKC3071. All mutations were introduced using the Quikchange Site-directed Mutagenesis (SDM) Kit (Stratagene) according to the manufacturer’s protocol. All introduced mutations were verified by DNA sequencing (MWG/Operon).

**Table 1:** Yeast strains used in this study

**Strain**	**Genotype**	**Source**
RSY335	*MAT***a***/MATα cyh2*^*r*^*-z ho::LYS2 leu2::hisG lys2 trp1::hisG ura3*	[63]
RSY562	*ama1::KANMX4*	[10]
RSY750	*AMA1-3HA CDC20-18MYC::URA3*	This study
RSY776	*MAT***a***cdh1::LEU2*	This study
RSY777	*cdh1::LEU2*	This study
RSY809	*cdc20-1*	[32]
RSY954	*cdc16-1*	This study
RSY1248	*MAT***a***CDC16::TAP mnd2::KANMX4*	This study
RSY1337	*MATα ade2 ade6 can1-100 his3-11,15 leu2-3,112 trp1-1 ura3-1 CDC27-9myc::LEU2*	This study
RSY1381	*MAT***a***CDC16::TAP::KAN/CDC16 mnd2::KANMX4 cdh1::LEU2*	This study
RSY1748	*MAT***a***CDC16::TAP/CDC16 mnd2::KANMX4 cdh1::LEU2 doc1:: TRP1*	This study
KCY328	*CDC27-9myc::LEU2*	This study
KCY419	*MATα mnd2::KANMX4*	This study
KCY456	*MAT***a***CDC16::TAP*	This study

*All strains, except RSY1337 are isogenic to RSY335. All strains are diploids and all alleles are homozygous unless indicated.

**Table 2:** Oligonucleotides used in this study with their accompanying mutation identified

**Name**	**Gene target**	**Mutation**	
**Created**	**Oligonucleotide**		
Db1	*AMA1*	RXXL-AXXA	ATTGTTGGTACAAAATTTGGCGCTATTCTTGCATATGATCAAAAAGAATTTTTTCATTCC
Db2	*AMA1*	RXXL-AXXA	TTCCCCATAAAAAACTGGAGTAAAGCACGTAAGGCCGAAGATGAAAATTTAATAGGATTGAAA
GXEN	*AMA1*	GXEN-GXAN	AAATTTTATGTTGGAGAGGCAAATGGCAATGTGAGCCTCTTTGAA

**Table 3:** Plasmids used in this study

**Mutation**	**Gene**	**Epitope tag**	**Plasmid name**	**Promotor**	**Type**	**References**
WT	*AMA1*	1 T7	pKC3036	*AMA1*	2 μ	[12]
CB	*AMA1*	1 T7	pKC3045	*AMA1*	2 μ	[12]
IR	*AMA1*	1 T7	pKC3046	*AMA1*	2 μ	[12]
CB/IR	*AMA1*	1 T7	pKC3048	*AMA1*	2 μ	[12]
Db1	*AMA1*	1 T7	pKC3126	*AMA1*	2 μ	This study
GxEN	*AMA1*	1 T7	pKC3123	*AMA1*	2 μ	This study
Db1/GXEN	*AMA1*	1 T7	pKC3127	*AMA1*	2 μ	This study
Db1/Db2/GXEN	*AMA1*	1 T7	pKC3129	*AMA1*	2 μ	This study
3HA	*AMA1*	3HA	pKC2057	*own*	Int	[10]
18Myc	*CDC20*	18Myc	pCdc20-myc18	*own*	Int	[65]
Codons 1-200	*AMA1*	GST	pKC3113	*GAL*	CEN	This study
Codons 1-200 CB	*AMA1*	GST	pKC3017	*GAL*	CEN	This study
9HA	*Clb1*	3HA	pKC427	*own*	CEN	[32]
3HA	*Clb5*	3HA	pKC440	*own*		This study
deletion	*Cdh1*	No tag	pWS176	*own*	Int.	[66]
Cb∆/IR∆	*AMA1*	no tag	pKC3095	*T7*	-	This study
Cb∆/IR∆/GXEN	*AMA1*	no tag	pKC3122	*T7*	-	This study
Cb∆/IR∆/GXEN/Db1	*AMA1*	no tag	pKC3124	*T7*	-	This study
Cb∆/IR∆/GXEN/Db1/Db2	*AMA1*	no tag	pKC3148	*T7*	-	This study
9Myc	*Cdc27*	9Myc	Cdc27-9Myc	*own*	int	P. Hieter
WT	*CDC20*	no Tag	pME41	*T7*	-	David Morgan
WT	*UBC4*	6HIS	6His-Ubc4	T7	-	Mark Solomon
-	*GST*	-	pEGKT	GAL1 *	2 μ	[67]
1-200CB	*GST-AMA1*	none	pKC3113	GAL1*	2 μ	[12]
1-200CB	*GST-AMA1*	none	pKC3017	GAL1*	2 μ	This study

* *CYC1* promoter driven by *GAL1* UAS.

### Meiotic and mitotic timecourse experiments

Growth and sporulation conditions were accomplished as previously described [63]. To permit *cdc20-1* and *cdc16-1* cultures to exit mitosis and enter the meiotic program, these cells were maintained at 23°C following transfer to sporulation medium for the amount of time indicated in the text before switching to the restrictive temperature (water bath). Quantitation of meiosis I and II was achieved by analyzing 4’, 6-diamidino-2-phenylindole (DAPI) stained cells as described [68]. A Nikon E800 fluorescence microscope was used for all experiments at a final magnification of 1000X. At least 200 cells were counted per timepoint. For the experiments using the galactose inducible GST expression constructs (Figure 4C), cells were grown to 1 × 10^7^ cells/ml in 2% raffinose, 2% galactose medium as previously described [69].

### Northern blot analysis, protein extract preparation, co-immunoprecipitation and Immunoblot analysis

Northern blot analysis was executed as previously described [32]. Protein extracts for co-immunoprecipitation and Western blot analyses (referred to as Immunoblot in text) were prepared as described [12]. Immunoblot analysis and co-immunoprecipitation experiments were conducted with 100 μg and 1 mg of soluble protein, respectively. Immunoblot signals were detected using goat anti-mouse secondary antibodies conjugated to alkaline phosphatase (Sigma) and the CDP-Star chemiluminescence kit (Tropix, Bedford, MA). Quantitation of Ama1p immunoblot signals from the mem brane was performed with an Image Station 4000R (Kodak Inc.) using Molecular Imaging Software (4.0.5) and standardized to tubulin. For all comparative immunoblot analyses, the membranes were treated with the same probe at the same time and the resulting signals were developed to the same extent.

### In vitro ubiquitylation assays

The in vitro ubiquitylation assays were performed as previously described [32,70]. In brief, the APC/C complex was purified from yeast extracts utilizing tandem affinity purification (TAP) tagged Cdc16p, a core component of this ubiquitin ligase. The ligase was incubated with *E. coli* produced ubiquitin conjugating enzyme (made from His_6_-Ubc4p (from M. Solomon) and in vitro transcription/translation produced Cdc20p. The Ama1p substrates were synthesized by in vitro transcription/translation (Promega) but in the presence of ^35^S-methionine. As previously described [70], 1 μl of the substrate was used per reaction (see Additional file 3 for input). The ubiquitylation reactions were conducted for the times indicated with fixed Cdc20p amounts (2.5 μl). The reactions were stopped by addition of 2X sample buffer and separated by SDS PAGE. The gels were fixed, soaked in Amplify® (Amersham Biosciences), then dried and subjected to autoradiography.

## Abbreviations

APC/C: Anaphase promoting complex; Db1: Destruction box (degron); GxEN: (destruction degron); CB: C-box (APC/C binding motif); IR: (APC/C binding motif); MI: Meiosis I; MII: Meiosis II; WT: Wild-type; SPM: Sporulation medium.

## Competing interests

The authors declare that they have no competing interests.

## Authors’ contributions

GT performed the experiments outlined in Figure 1A, B and C, 2 and 3A and B. RL performed the experiments outlined in Figure 4. MM performed the experiment in Figure 1D and 3C. KFC and RS wrote the manuscript. All authors read and approved the final manuscript.

## Supplementary Material

Additional file 1 **Analysis of *cdc20-1 *during meiosis.** A: Northern blot analysis of *cdc20-1* cells progressing through meiosis at 23°C showing the expression of early (*IME2*), early middle (*NDT80*), middle (*SPS4*) and late genes (*SPS100*). *ENO1* represents the loading control. Click here for file

Additional file 2 **Cdh1p is not required to degrade Ama1p during meiosis. A:** Fluorescence and Nomarski (Nom.) images (1000X magnification) of DAPI stained wild type (RSY335) and *cdh1∆* (RSY777) diploids 24 h after transfer to sporulation medium. **B:** Rate of appearance of bi- and tetranucleated cells in wild type and *cdh1∆* cells after entry into the meiotic program. Percentage of cells in the culture executing at least one meiotic division, presented as a function of time following transfer to sporulation medium. MI, Meiosis I; MII meiosis II. **C:*** %* mono, bi and tetranucleated cells in the total population after 24 h in sporulation medium. **D:***cdh1∆* strain (RSY777) harboring Ama1p-T7 (pKC3036) was induced to enter meiosis and timepoints taken as indicated. Immunoblot analysis of immunoprecipitated protein extracts was conducted to detect Ama1p-T7. Immunoblot analysis of Tub1p was used as a loading control. **E:** Viability of wild type (RSY335) and *cdh1∆* (RSY777) tetrad spores. Click here for file

Additional file 3 ^**35**^**S labeled Ama1p input for ubiquitylation assays.** 1 μl of ^35^S labeled in vitro transcription/translation Ama1p prepared from either pKC3095 (lane 1), pKC3122 (lane 2) pKC3148 (lane 3) or pKC3124 (lane 4) or zero DNA control was visualized by autoradiography. Click here for file

## References

[B1] Herskowitz I Life style of the budding yeast *Saccharomyces cerevisiae* Microbiol Rev 1988 52 536 553 3070323 10.1128/mr.52.4.536-553.1988 PMC373162

[B2] Vershon A Pierce M Transcriptional regulation of meiosis in yeast Curr Opin Cell Biol 2000 12 334 339 10.1016/S0955-0674(00)00104-6 10801467

[B3] Thornton BR Ng TM Matyskiela ME Carroll CW Morgan DO Toczyski DP An architectural map of the anaphase-promoting complex Genes Dev 2006 20 449 460 10.1101/gad.1396906 16481473 PMC1369047

[B4] Yu H Cdc20: a WD40 activator for a cell cycle degradation machine Mol Cell 2007 27 3 16 10.1016/j.molcel.2007.06.009 17612486

[B5] Barford D Structural insights into anaphase-promoting complex function and mechanism Philos Trans R Soc Lond B Biol Sci 2011 366 3605 3624 10.1098/rstb.2011.0069 22084387 PMC3203452

[B6] Foe IT Foster SA Cheung SK DeLuca SZ Morgan DO Toczyski DP Ubiquitination of Cdc20 by the APC occurs through an intramolecular mechanism Curr Biol 2011 21 1870 1877 10.1016/j.cub.2011.09.051 22079111 PMC3430386

[B7] Pesin JA Orr-Weaver TL Regulation of APC/C activators in mitosis and meiosis Annu Rev Cell Dev Biol 2008 24 475 499 10.1146/annurev.cellbio.041408.115949 18598214 PMC4070676

[B8] Cooper KF Strich R Meiotic control of the APC/C: similarities & differences from mitosis Cell Div 2011 6 16 10.1186/1747-1028-6-16 21806783 PMC3162515

[B9] Chu S DeRisi J Eisen M Mulholland J Botstein D Brown PO Herskowitz I The transcriptional program of sporulation in budding yeast Science 1998 282 699 705 9784122 10.1126/science.282.5389.699

[B10] Cooper KF Egeland DE Mallory MJ Jarnik M Strich R Ama1p is a Meiosis-Specific Regulator of the Anaphase Promoting Complex/Cyclosome in yeast Proc. Natl. Acad. Sci. USA 2000 97 14548 14553 10.1073/pnas.250351297 11114178 PMC18956

[B11] Diamond AE Park JS Inoue I Tachikawa H Neiman AM The anaphase promoting complex targeting subunit Ama1 links meiotic exit to cytokinesis during sporulation in Saccharomyces cerevisiae Mol Biol Cell 2009 20 134 145 10.1091/mbc.E08-06-0615 18946082 PMC2613089

[B12] Tan GS Magurno J Cooper KF Ama1p-activated anaphase-promoting complex regulates the destruction of Cdc20p during meiosis II Mol Biol Cell 2011 22 315 326 10.1091/mbc.E10-04-0360 21118994 PMC3031463

[B13] Rabitsch KP Toth A Galova M Schleiffer A Schaffner G Aigner E Rupp C Penkner AM Moreno-Borchart AC Primig M A screen for genes required for meiosis and spore formation based on whole-genome expression Curr Biol 2001 11 1001 1009 10.1016/S0960-9822(01)00274-3 11470404

[B14] McDonald CM Cooper KF Winter E The Ama1-Directed Anaphase-Promoting Complex Regulates the Smk1 Mitogen-Activated Protein Kinase During Meiosis in Yeast Genetics 2005 171 901 911 10.1534/genetics.105.045567 16079231 PMC1456836

[B15] Coluccio A Bogengruber E Conrad MN Dresser ME Briza P Neiman AM Morphogenetic pathway of spore wall assembly in Saccharomyces cerevisiae Eukaryot Cell 2004 3 1464 1475 10.1128/EC.3.6.1464-1475.2004 15590821 PMC539034

[B16] Kamieniecki RJ Liu L Dawson DS FEAR but not MEN genes are required for exit from meiosis I Cell Cycle 2005 4 1093 1098 15970684

[B17] Glotzer M Murray AW Kirschner MW Cyclin is degraded by the ubiquitin pathway Nature 1991 349 132 138 10.1038/349132a0 1846030

[B18] Pfleger CM Lee E Kirschner MW Substrate recognition by the Cdc20 and Cdh1 components of the anaphase-promoting complex Genes Dev 2001 15 2396 2407 10.1101/gad.918201 11562349 PMC312782

[B19] Buschhorn BA Petzold G Galova M Dube P Kraft C Herzog F Stark H Peters JM Substrate binding on the APC/C occurs between the coactivator Cdh1 and the processivity factor Doc1 Nat Struct Mol Biol 2011 18 6 13 10.1038/nsmb.1979 21186364 PMC4300845

[B20] Barford D Structure, function and mechanism of the anaphase promoting complex (APC/C) Q Rev Biophys 2011 44 153 190 10.1017/S0033583510000259 21092369

[B21] King EM van der Sar SJ Hardwick KG Mad3 KEN boxes mediate both Cdc20 and Mad3 turnover, and are critical for the spindle checkpoint PLoS One 2007 2 e342 10.1371/journal.pone.0000342 17406666 PMC1829190

[B22] Bolte M Dieckhoff P Krause C Braus GH Irniger S Synergistic inhibition of APC/C by glucose and activated Ras proteins can be mediated by each of the Tpk1-3 proteins in Saccharomyces cerevisiae Microbiology 2003 149 1205 1216 10.1099/mic.0.26062-0 12724382

[B23] Yamano H Gannon J Mahbubani H Hunt T Cell cycle-regulated recognition of the destruction box of cyclin B by the APC/C in Xenopus egg extracts Mol Cell 2004 13 137 147 10.1016/S1097-2765(03)00480-5 14731401

[B24] Da Fonseca PC Kong EH Zhang Z Schreiber A Williams MA Morris EP Barford D Structures of APC/C(Cdh1) with substrates identify Cdh1 and Apc10 as the D-box co-receptor Nature 2011 470 274 278 10.1038/nature09625 21107322 PMC3037847

[B25] Passmore LA McCormack EA Au SW Paul A Willison KR Harper JW Barford D Doc1 mediates the activity of the anaphase-promoting complex by contributing to substrate recognition EMBO J 2003 22 786 796 10.1093/emboj/cdg084 12574115 PMC145444

[B26] Burton JL Tsakraklides V Solomon MJ Assembly of an APC-Cdh1-substrate complex is stimulated by engagement of a destruction box Mol Cell 2005 18 533 542 10.1016/j.molcel.2005.04.022 15916960

[B27] Kraft C Vodermaier HC Maurer-Stroh S Eisenhaber F Peters JM The WD40 propeller domain of Cdh1 functions as a destruction box receptor for APC/C substrates Mol Cell 2005 18 543 553 10.1016/j.molcel.2005.04.023 15916961

[B28] Izawa D Pines J How APC/C-Cdc20 changes its substrate specificity in mitosis Nat Cell Biol 2011 13 223 233 10.1038/ncb2165 21336306 PMC3059483

[B29] Wendt KS Vodermaier HC Jacob U Gieffers C Gmachl M Peters JM Huber R Sondermann P Crystal structure of the APC10/DOC1 subunit of the human anaphase-promoting complex Nat Struct Biol 2001 8 784 788 10.1038/nsb0901-784 11524682

[B30] Carroll CW Enquist-Newman M Morgan DO The APC subunit Doc1 promotes recognition of the substrate destruction box Curr Biol 2005 15 11 18 10.1016/j.cub.2004.12.066 15649358

[B31] Lamb JR Michaud WA Sikorski RS Hieter PA Cdc16p, Cdc23p and Cdc27p form a complex essential for mitosis EMBO J 1994 13 4321 4328 7925276 10.1002/j.1460-2075.1994.tb06752.x PMC395359

[B32] Mallory MJ Cooper KF Strich R Meiosis-specific destruction of the Ume6p repressor by the Cdc20-directed APC/C Mol Cell 2007 27 951 961 10.1016/j.molcel.2007.08.019 17889668 PMC2034308

[B33] Oelschlaegel T Schwickart M Matos J Bogdanova A Camasses A Havlis J Shevchenko A Zachariae W The yeast APC/C subunit Mnd2 prevents premature sister chromatid separation triggered by the meiosis-specific APC/C-Ama1 Cell 2005 120 773 788 10.1016/j.cell.2005.01.032 15797379

[B34] Hepworth SR Ebisuzaki LK Segall J A 15-base-pair element activates the SPS4 gene midway through sporulation in Saccharomyces cerevisiae Mol Cell Biol 1995 15 3934 3944 7791799 10.1128/mcb.15.7.3934 PMC230633

[B35] Law DTS Segall J The SPS100 gene of Saccharomyces cerevisiae is activated late in the sporulation process and contributes to spore wall maturation Mol Cell Biol 1988 8 912 922 3280971 10.1128/mcb.8.2.912-922.1988 PMC363223

[B36] Harper JW Burton JL Solomon MJ The anaphase-promoting complex: it's not just for mitosis any more Genes Dev 2002 16 2179 2206 10.1101/gad.1013102 12208841

[B37] Schwab M Neutzner M Mocker D Seufert W Yeast Hct1 recognizes the mitotic cyclin Clb2 and other substrates of the ubiquitin ligase APC EMBO J 2001 20 5165 5175 10.1093/emboj/20.18.5165 11566880 PMC125620

[B38] Robbins JA Cross FR Regulated degradation of the APC coactivator Cdc20 Cell Div 2010 5 23 10.1186/1747-1028-5-23 20831816 PMC2949745

[B39] Foster SA Morgan DO The APC/C subunit Mnd2/Apc15 promotes Cdc20 autoubiquitination and spindle assembly checkpoint inactivation Mol Cell 2012 47 921 932 10.1016/j.molcel.2012.07.031 22940250 PMC3462283

[B40] Pesin JA Orr-Weaver TL Developmental role and regulation of cortex, a meiosis-specific anaphase-promoting complex/cyclosome activator PLoS Genet 2007 3 e202 10.1371/journal.pgen.0030202 18020708 PMC2077894

[B41] Rudner AD Murray AW Phosphorylation by cdc28 activates the Cdc20-dependent activity of the anaphase-promoting complex J Cell Biol 2000 149 1377 1390 10.1083/jcb.149.7.1377 10871279 PMC2175139

[B42] Tang Z Shu H Oncel D Chen S Yu H Phosphorylation of Cdc20 by Bub1 provides a catalytic mechanism for APC/C inhibition by the spindle checkpoint Mol Cell 2004 16 387 397 10.1016/j.molcel.2004.09.031 15525512

[B43] Chung E Chen RH Phosphorylation of Cdc20 is required for its inhibition by the spindle checkpoint Nat Cell Biol 2003 5 748 753 10.1038/ncb1022 12855955

[B44] Labit H Fujimitsu K Bayin NS Takaki T Gannon J Yamano H Dephosphorylation of Cdc20 is required for its C-box-dependent activation of the APC/C EMBO J 2012 31 3351 3362 10.1038/emboj.2012.168 22713866 PMC3411074

[B45] Reimann JD Freed E Hsu JY Kramer ER Peters JM Jackson PK Emi1 is a mitotic regulator that interacts with Cdc20 and inhibits the anaphase promoting complex Cell 2001 105 645 655 10.1016/S0092-8674(01)00361-0 11389834

[B46] Martinez JS Jeong DE Choi E Billings BM Hall MC Acm1 is a negative regulator of the CDH1-dependent anaphase-promoting complex/cyclosome in budding yeast Mol Cell Biol 2006 26 9162 9176 10.1128/MCB.00603-06 17030612 PMC1698549

[B47] Choi E Dial JM Jeong DE Hall MC Unique D box and KEN box sequences limit ubiquitination of Acm1 and promote pseudosubstrate inhibition of the anaphase-promoting complex J Biol Chem 2008 283 23701 23710 10.1074/jbc.M803695200 18596038 PMC3259782

[B48] Ostapenko D Burton JL Wang R Solomon MJ Pseudosubstrate inhibition of the anaphase-promoting complex by Acm1: regulation by proteolysis and Cdc28 phosphorylation Mol Cell Biol 2008 28 4653 4664 10.1128/MCB.00055-08 18519589 PMC2493364

[B49] Enquist-Newman M Sullivan M Morgan DO Modulation of the mitotic regulatory network by APC-dependent destruction of the Cdh1 inhibitor Acm1 Mol Cell 2008 30 437 446 10.1016/j.molcel.2008.04.004 18498748 PMC2494983

[B50] Burton JL Solomon MJ Mad3p, a pseudosubstrate inhibitor of APCCdc20 in the spindle assembly checkpoint Genes Dev 2007 21 655 667 10.1101/gad.1511107 17369399 PMC1820940

[B51] Sczaniecka M Feoktistova A May KM Chen JS Blyth J Gould KL Hardwick KG The spindle checkpoint functions of Mad3 and Mad2 depend on a Mad3 KEN box-mediated interaction with Cdc20-anaphase-promoting complex (APC/C) J Biol Chem 2008 283 23039 23047 10.1074/jbc.M803594200 18556659 PMC2516979

[B52] Lara-Gonzalez P Scott MI Diez M Sen O Taylor SS BubR1 blocks substrate recruitment to the APC/C in a KEN-box-dependent manner J Cell Sci 2011 124 4332 4345 10.1242/jcs.094763 22193957 PMC3258114

[B53] Jaquenoud M Van Drogen F Peter M Cell cycle-dependent nuclear export of Cdh1p may contribute to the inactivation of APC/C(Cdh1) EMBO J 2002 21 6515 6526 10.1093/emboj/cdf634 12456658 PMC136938

[B54] Schmidt A Duncan PI Rauh NR Sauer G Fry AM Nigg EA Mayer TU Xenopus polo-like kinase Plx1 regulates XErp1, a novel inhibitor of APC/C activity Genes Dev 2005 19 502 513 10.1101/gad.320705 15713843 PMC548950

[B55] Kimata Y Kitamura K Fenner N Yamano H Mes1 controls the meiosis I to meiosis II transition by distinctly regulating the anaphase-promoting complex/cyclosome coactivators Fzr1/Mfr1 and Slp1 in fission yeast Mol Biol Cell 2011 22 1486 1494 10.1091/mbc.E10-09-0774 21389117 PMC3084671

[B56] Hwang LH Murray AW A novel yeast screen for mitotic arrest mutants identifies DOC1, a new gene involved in cyclin proteolysis Mol Biol Cell 1997 8 1877 1887 10.1091/mbc.8.10.1877 9348530 PMC25633

[B57] Passmore LA Barford D Coactivator functions in a stoichiometric complex with anaphase-promoting complex/cyclosome to mediate substrate recognition EMBO Rep 2005 6 873 878 10.1038/sj.embor.7400482 16113654 PMC1369160

[B58] Matyskiela ME Morgan DO Analysis of activator-binding sites on the APC/C supports a cooperative substrate-binding mechanism Mol Cell 2009 34 68 80 10.1016/j.molcel.2009.02.027 19362536 PMC2754851

[B59] Schreiber A Stengel F Zhang Z Enchev RI Kong EH Morris EP Robinson CV Da Fonseca PC Barford D Structural basis for the subunit assembly of the anaphase-promoting complex Nature 2011 470 227 232 10.1038/nature09756 21307936

[B60] Passmore LA Booth CR Venien-Bryan C Ludtke SJ Fioretto C Johnson LN Chiu W Barford D Structural analysis of the anaphase-promoting complex reveals multiple active sites and insights into polyubiquitylation Mol Cell 2005 20 855 866 10.1016/j.molcel.2005.11.003 16364911

[B61] Cooper KF Mallory MJ Strich R Oxidative stress-induced destruction of the yeast C-type cyclin Ume3p requires the Phosphatidylinositol-specific phospholipase C and the 26S proteasome Mol Cell Biol 1999 19 3338 3348 10207058 10.1128/mcb.19.5.3338 PMC84127

[B62] Cooper KF Mallory MJ Guacci V Lowe K Strich R Pds1p is required for meiotic recombination and prophase I progression in Saccharomyces cerevisiae Genetics 2009 181 65 79 19001291 10.1534/genetics.108.095513 PMC2621190

[B63] Cooper KF Mallory MJ Smith JS Strich R Stress and developmental regulation of the yeast C-type cyclin *UME3* (*SRB11*/*SSN8*) EMBO J 1997 16 4665 4675 10.1093/emboj/16.15.4665 9303311 PMC1170093

[B64] Longtine MS McKenzie AR Demarini DJ Shah NG Wach A Brachat A Philippsen P Pringle JR Additional modules for versatile and economical PCR-based gene deletion and modification Saccharomyces cerevisiae 1998 Yeast 14 953 961 10.1002/(SICI)1097-0061(199807)14:10<953::AID-YEA293>3.0.CO;2-U 9717241

[B65] Shirayama M Zachariae W Ciosk R Nasmyth K The Polo-like kinase Cdc5p and the WD-repeat protein Cdc20p/fizzy are regulators and substrates of the anaphase promoting complex in *Saccharomyces cerevisiae* EMBO J 1998 17 1336 1349 10.1093/emboj/17.5.1336 9482731 PMC1170482

[B66] Schwab M Neutzer M Mocker D Seufert W Yeast Hct1 recognises the mitotic Clb2 and other substrates of the ubiquitin ligase APC EMBO 2001 20 5165 5175 10.1093/emboj/20.18.5165 PMC125620 11566880

[B67] Burton JL Solomon MJ D box and KEN box motifs in budding yeast Hsl1p are required for APC-mediated degradation and direct binding to Cdc20p and Cdh1p Genes Dev 2001 15 2381 2395 10.1101/gad.917901 11562348 PMC312781

[B68] Cooper KF Strich R Saccharomyces cerevisiae C-type cyclin Ume3p/Srb11p is required for efficient induction and execution of meiotic development Eukaryot Cell 2002 1 66 74 10.1128/EC.01.1.66-74.2002 12455972 PMC118056

[B69] Prinz S Hwang ES Visintin R Amon A The regulation of Cdc20 proteolysis reveals a role for APC components Cdc23 and Cdc27 during S phase and early mitosis Curr Biol 1998 8 750 760 10.1016/S0960-9822(98)70298-2 9651679

[B70] Passmore LA McCormack EA Au SWN Paul A Willison KR Harper JW Barford D Doc1 mediates the activity of the anaphase promoting complex by contributing to substrate recognition EMBO 2003 22 786 796 10.1093/emboj/cdg084 PMC145444 12574115

