# Supplementary figures and images for: Mutually dependent degradation of Ama1p and Cdc20p terminates APC/C ubiquitin ligase activity at the completion of meiotic development in yeast
Source: Cell Div. 2013 Jul 1;8:9. doi: 10.1186/1747-1028-8-9 (PMC3734102; doi:10.1186/1747-1028-8-9)

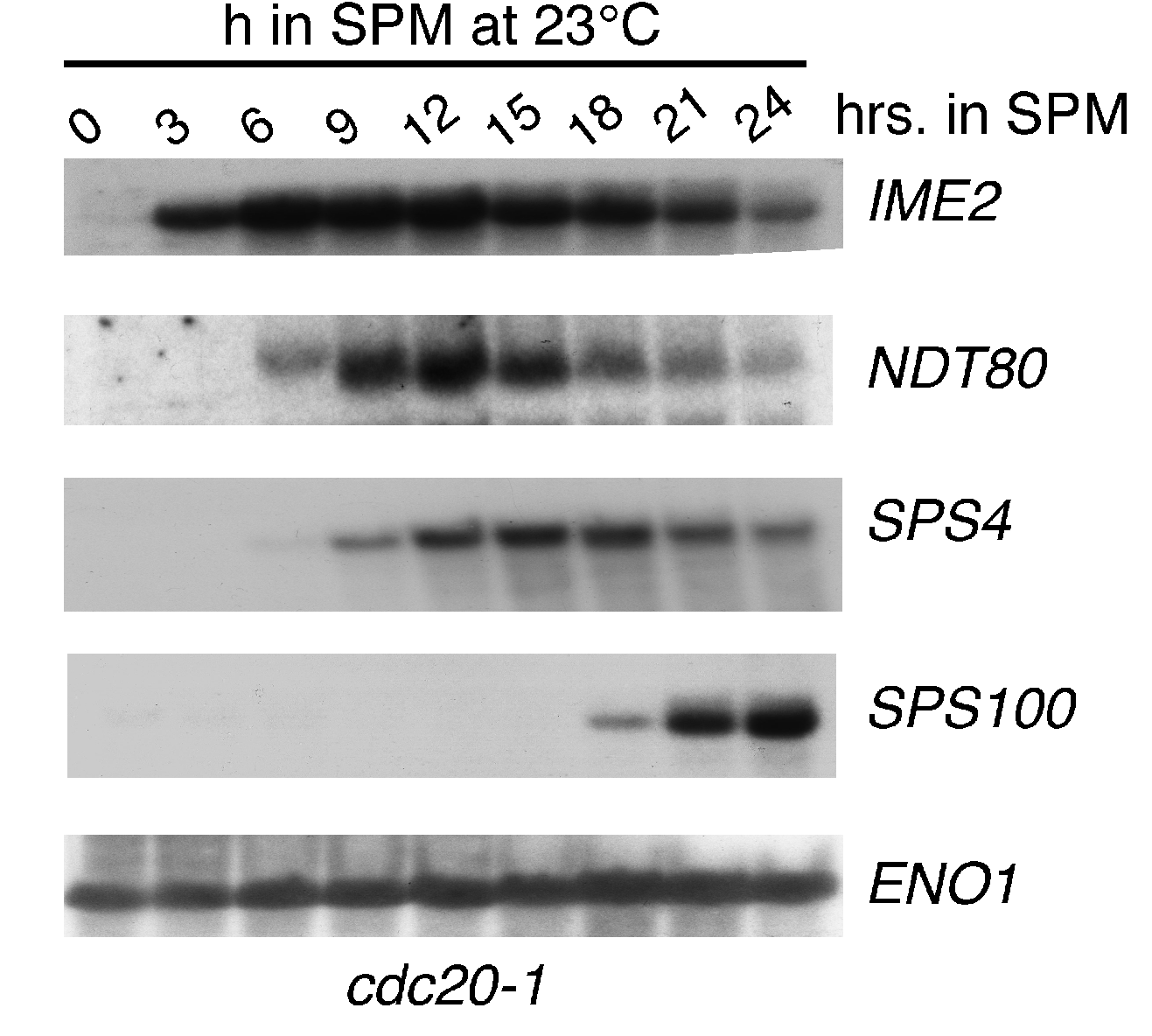

Supplement: Additional file 1 — Analysis of cdc20-1 during meiosis. A: Northern blot analysis of cdc20-1 cells progressing through meiosis at 23°C showing the expression of early (IME2), early middle (NDT80), middle (SPS4) and late genes (SPS100). ENO1 represents the loading control. [file 1747-1028-8-9-S1.tiff]

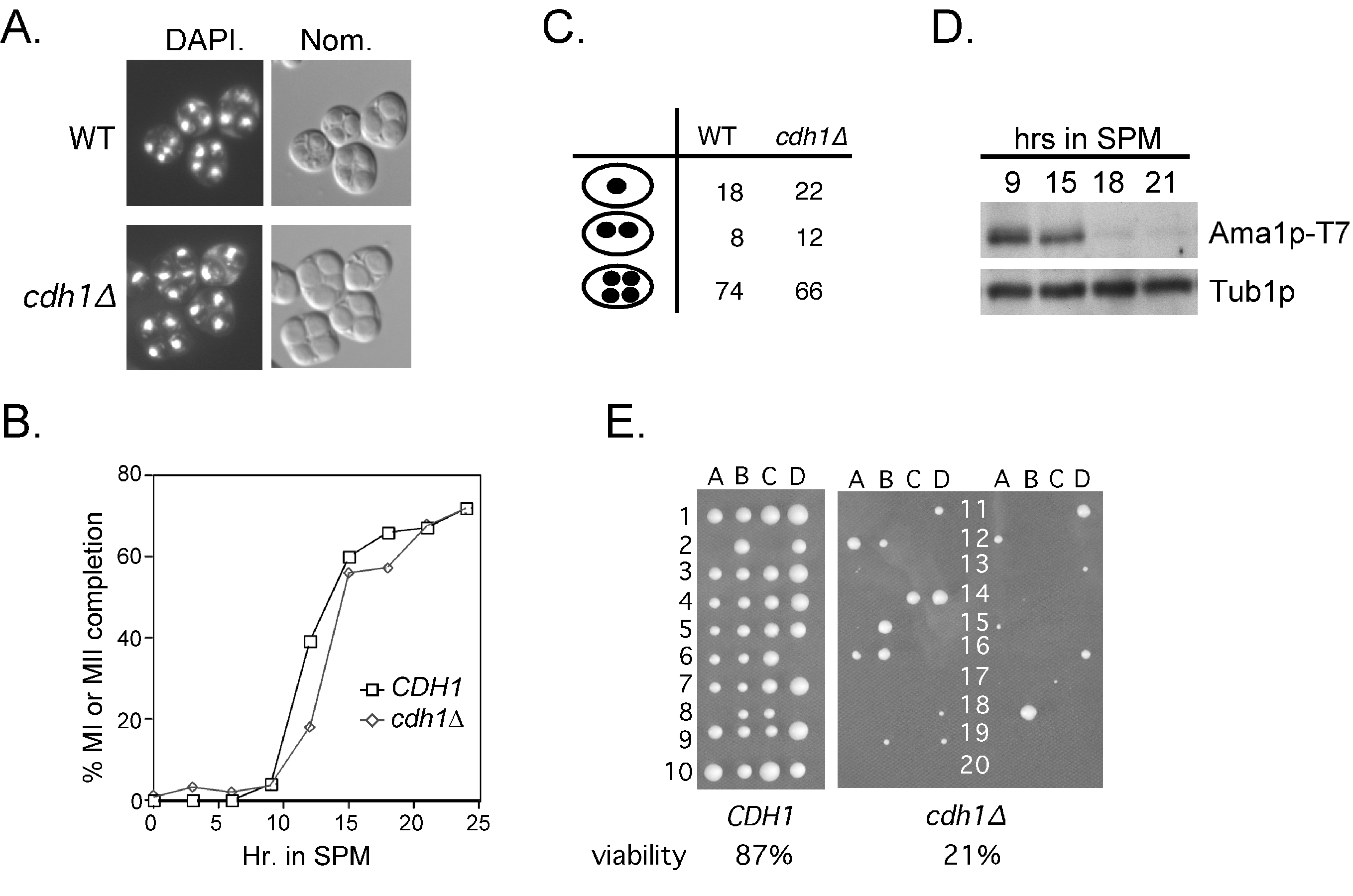

Supplement: Additional file 2 — Cdh1p is not required to degrade Ama1p during meiosis. A: Fluorescence and Nomarski (Nom.) images (1000X magnification) of DAPI stained wild type (RSY335) and cdh1∆ (RSY777) diploids 24 h after transfer to sporulation medium. B: Rate of appearance of bi- and tetranucleated cells in wild type and cdh1∆ cells after entry into the meiotic program. Percentage of cells in the culture executing at least one meiotic division, presented as a function of time following transfer to sporulation medium. MI, Meiosis I; MII meiosis II. C: % mono, bi and tetranucleated cells in the total population after 24 h in sporulation medium. D:cdh1∆ strain (RSY777) harboring Ama1p-T7 (pKC3036) was induced to enter meiosis and timepoints taken as indicated. Immunoblot analysis of immunoprecipitated protein extracts was conducted to detect Ama1p-T7. Immunoblot analysis of Tub1p was used as a loading control. E: Viability of wild type (RSY335) and cdh1∆ (RSY777) tetrad spores. [file 1747-1028-8-9-S2.tiff]

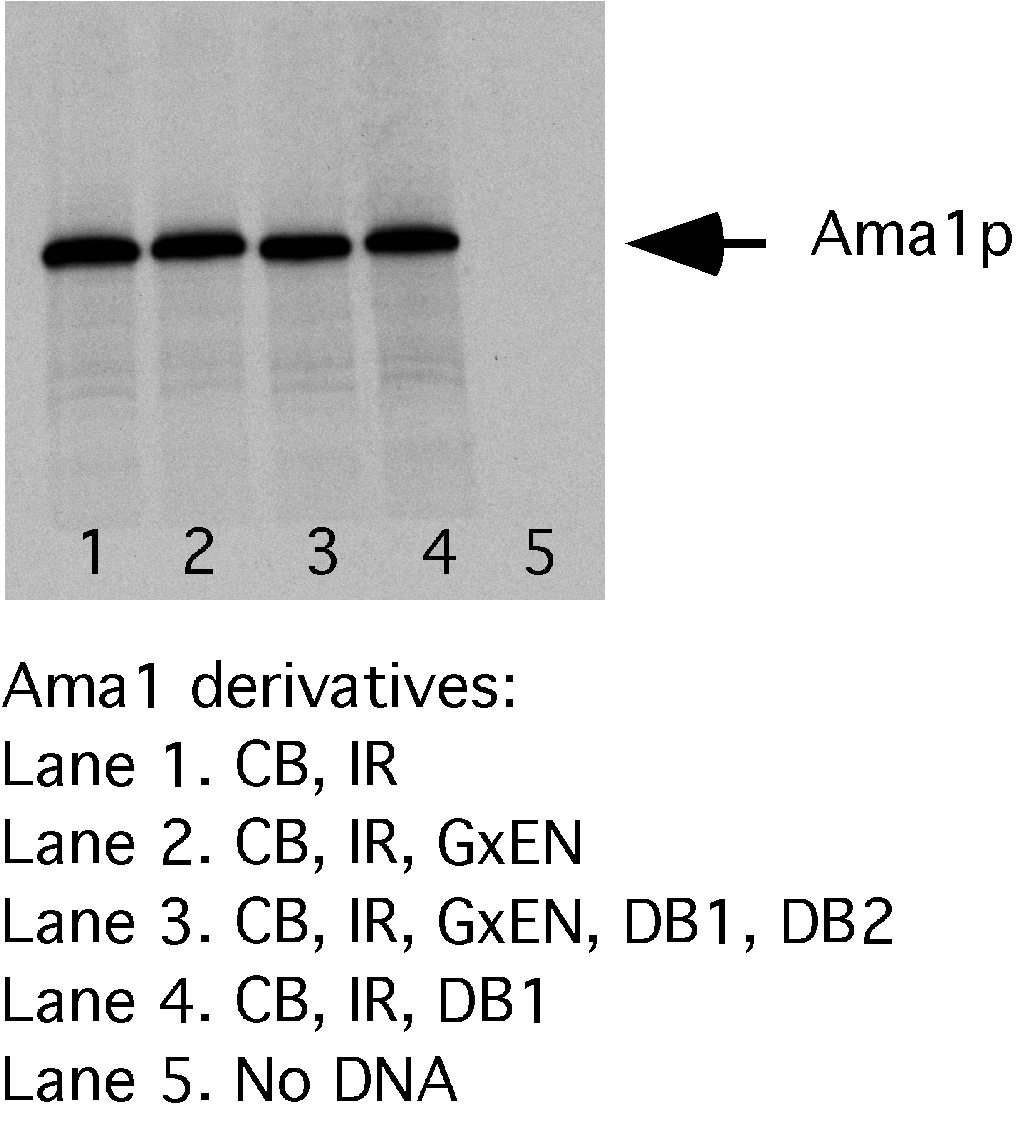

Supplement: Additional file 3 — 35S labeled Ama1p input for ubiquitylation assays. 1 μl of 35S labeled in vitro transcription/translation Ama1p prepared from either pKC3095 (lane 1), pKC3122 (lane 2) pKC3148 (lane 3) or pKC3124 (lane 4) or zero DNA control was visualized by autoradiography. [file 1747-1028-8-9-S3.tiff]
